# Consumption‐Based Conservation Targeting: Linking Biodiversity Loss to Upstream Demand through a Global Wildlife Footprint

**DOI:** 10.1111/con4.12321

**Published:** 2016-11-09

**Authors:** Justin Kitzes, Eric Berlow, Erin Conlisk, Karlheinz Erb, Katsunori Iha, Neo Martinez, Erica A. Newman, Christoph Plutzar, Adam B. Smith, John Harte

**Affiliations:** ^1^ Energy and Resources Group University of California Berkeley CA 94720 USA; ^2^ Vibrant Data Inc. San Francisco CA 94108 USA; ^3^ Lawrence Berkeley National Lab Berkeley CA 94720 USA; ^4^ Institute of Social Ecology Vienna (SEC) Alpen‐Adria Universitaet Klagenfurt ‐ Wien ‐ Graz Vienna Austria; ^5^ Global Footprint Network Oakland CA 94607 USA; ^6^ Pacfic Ecoinformatics and Computational Ecology Lab Berkeley CA 94703 USA; ^7^ Energy and Resources Group and Department of Environmental Science, Policy, and Management University of California Berkeley CA 94720 USA; ^8^ Missouri Botanical Garden St. Louis MO 63110 USA

**Keywords:** Biodiversity, consumption, economics, footprint, GIS, human ecology, input output, land use, life cycle, spatial

## Abstract

Although most conservation efforts address the direct, local causes of biodiversity loss, effective long‐term conservation will require complementary efforts to reduce the upstream economic pressures, such as demands for food and forest products, which ultimately drive these downstream losses. Here, we present a wildlife footprint analysis that links global losses of wild birds to consumer purchases across 57 economic sectors in 129 regions. The United States, India, China, and Brazil have the largest regional wildlife footprints, while per‐person footprints are highest in Mongolia, Australia, Botswana, and the United Arab Emirates. A US$100 purchase of bovine meat or rice products occupies approximately 0.1 km^2^ of wild bird ranges, displacing 1–2 individual birds, for 1 year. Globally significant importer regions, including Japan, the United Kingdom, Germany, Italy, and France, have large footprints that drive wildlife losses elsewhere in the world and represent important targets for consumption‐focused conservation attention.

## Introduction

Despite growing recognition of the importance of biodiversity to natural and human communities, global biodiversity loss continues at a rapid pace (Butchart *et al*. 2010). In response, great progress has been made in identifying the geographic locations where biodiversity loss is occurring and the proximate causes of those losses, such as land use change, climate change, invasive species, overexploitation, and pollution (Sanderson *et al*. 2002; Millennium Ecosystem Assessment 2005; IUCN 2015; McCauley *et al*. 2015; Newbold *et al*. 2015).

These proximate causes of biodiversity loss, however, are themselves driven by upstream economic activities, particularly demand for ecosystem goods and services, which may be geographically distant from the locations of biodiversity loss. Palm oil plantations that threaten orangutan populations in southeast Asia, for example, continue to expand largely to provide palm oil for use in food and consumer products elsewhere in the world (Clay 2004; Koh & Wilcove 2007; Nellemann *et al*. 2007). While a traditional conservation perspective would focus on the local communities clearing land for plantations, an upstream consumption‐based perspective would focus attention on the end consumers whose purchasing decisions ultimately drive this land clearing.

A key method for linking downstream environmental impacts to upstream drivers is “footprint” analysis, a widely used environmental accounting technique in human and industrial ecology. Beginning with the land‐based ecological footprint (Wackernagel & Rees 1998; Wackernagel *et al*. 2002; Kitzes *et al*. 2009), footprint methods have been applied to analyze carbon (Hertwich & Peters 2009; Davis & Caldeira 2010), water (Hoekstra & Mekonnen 2012), nitrogen (Leach *et al*. 2012; Oita *et al*. 2016), and biodiversity threats (Lenzen *et al*. 2012; Moran *et al*. 2016; Moran & Kanemoto 2017). Closely related to life cycle assessment, footprints evaluate the total impacts of an economic activity, such as the purchase of a consumer product, wherever they occur in the world or in a supply chain.

Lenzen *et al*. (2012) presented the first comprehensive global footprint analysis for biodiversity. This study evaluated global trade in animal “species threats” based on data from the International Union for Conservation of Nature (IUCN) Red List. These data, however, exclude any effects of human activities on nonthreatened species and ignore potentially substantial effects on species populations or ranges that do not reach a threshold of threatening a species with extinction. The unit of summed “species threats” must also be translated into changes in population sizes or range extents for comparison with other studies that measure biodiversity impacts using these more common metrics.

More recently, Chaudhary *et al*. (2015), Chaudhary *et al*. (2016), and Chaudhary & Kastner (2016) used the countryside species–area relationship to estimate global and regional species loss due to land conversion and international trade. The power‐law form of the species–area relationship used in these studies, however, has been called into question on both empirical and theoretical grounds (Harte *et al*. 2009; Wilber *et al*. 2015). Additionally, many of the potential extinctions estimated in these studies may not yet be realized, and it is unclear how extinctions would be allocated to different actors across time or how this temporal dimension affects the possibility of conservation intervention.

Building on these prior analyses, this manuscript develops a global wildlife footprint that links global land use‐driven impacts on wild bird species to consumer purchases in 57 economic sectors across 129 geographic regions. The wildlife footprint evaluates impacts on all terrestrial breeding bird species and presents results in the relatively interpretable units of “occupied bird ranges” and “missing individual birds” (see *Methods*). These metrics provide a more sensitive measure of human impacts on biodiversity than measures of species extinction (Ehrlich & Daily 1993; Ceballos & Ehrlich 2002) and incorporate changes in the status of common as well as rare species (Gaston & Fuller 2008; Gaston 2010).

## Methods

This analysis uses two metrics to calculate the global wildlife footprint: occupied bird ranges and missing individual birds. The occupied bird ranges metric begins with a count of the number of present‐day breeding bird ranges that overlap a geographic area. Global maps of bird ranges were obtained from BirdLife International and NatureServe (Bird Species Distribution Maps of the World 2015, v5.0), and range polygons were filtered to those representing extant or probably extant presence, native, reintroduced, or introduced origin, and resident or breeding seasonality. These polygons were rasterized and summed to create a map of the number of present‐day breeding bird ranges overlapping each grid cell.

The missing individual birds metric begins with the number of wild breeding birds of any species that would be present in the intact potential vegetation cover in a geographic area. A global map of potential climatically driven vegetation classes was obtained from Ramankutty & Foley (1999). Each of the 15 vegetation classes in this map was assigned a baseline bird density based on surveys of breeding bird density in each vegetation type (Gaston *et al*. 2003). Medium bird densities from Gaston *et al*. (2003) were used to calculate central estimates of baseline bird counts, local losses, and wildlife footprints. Lower and upper bounds around these central estimates were calculated using low‐ and high‐density estimates from this same source.

These baseline wildlife maps were then combined with a map of the human appropriation of net primary productivity (HANPP), which was used as a measure of the fractional losses of wildlife occurring in each grid cell. Following Haberl *et al*. (2007), HANPP was defined as one minus the ratio of the net primary productivity found in a grid cell after human uses and the net primary productivity that would be available in the grid cell in the absence of human land use or harvest activities. The global HANPP map of Haberl *et al*. (2007) was disaggregated into four major human land uses: cropland, pasture and grazing, forestry, and built up land. These HANPP maps were multiplied by the fractional area of each land use per grid cell (Erb *et al*. 2007) and the baseline wildlife maps to estimate bird losses occurring in each grid cell. Losses due to cropland were further divided between 13 crop groups using data from Monfreda *et al*. (2008).

Global multiregional input‐output tables were extracted from the Global Trade Analysis Project (GTAP) v8a database (Peters *et al*. 2011) and used to link wildlife losses in grid cells to upstream consumer demand (Miller & Blair 2009; Kitzes 2013). Within each region, wildlife losses were allocated across 57 economic sectors according to the proximate causes of wildlife losses within each region. The resulting direct input vector measures the wildlife losses driven directly by each of 57 sectors within 129 regions. Local wildlife losses for each region were calculated as the sum of the direct input vector within each region.

The direct input vector and multiregional input‐output table were used to calculate the total downstream wildlife losses driven by consumer purchases from each of 57 economic sectors (a sector footprint) and within each of 129 regions (a regional footprint). A per capita regional footprint was calculated by dividing a regional footprint by that region's human population.

A region's footprint may drive downstream wildlife losses outside of its own borders that are then “imported” into the region embodied in imports of goods and services. Conversely, regions may “export” portions of their local wildlife losses through the export of goods and services to other regions. To identify regions that are key players in the global wildlife footprint trade, regions that were above the 66th‐percentile in both regional footprint and the proportion of footprint that was imported, as measured by both the occupied range and missing individuals metrics, were defined as globally significant importing regions. Globally significant exporting regions were defined as those regions that were above the 66th‐percentile in both local losses and the proportion of these local losses that were exported.

Additional details on our data sources and analytical methods can be found in *Appendix A*.

## Results

In units of bird ranges, the total global wildlife footprint is estimated as 4.3 billion km^2^ of occupied bird ranges, representing 20% of the 21.7 billion km^2^ of extant terrestrial bird breeding ranges. In units of individuals, the global wildlife footprint is estimated as 26 ± 13 billion individual missing birds, representing 21% of the 120 ± 59 billion breeding birds that would be present on the planet in the absence of human land use and harvest activities. The average human on the planet occupies 0.65 km^2^ of breeding bird ranges and displaces 3.9 ± 1.9 individual breeding birds on an ongoing basis.

**Figure 1:**
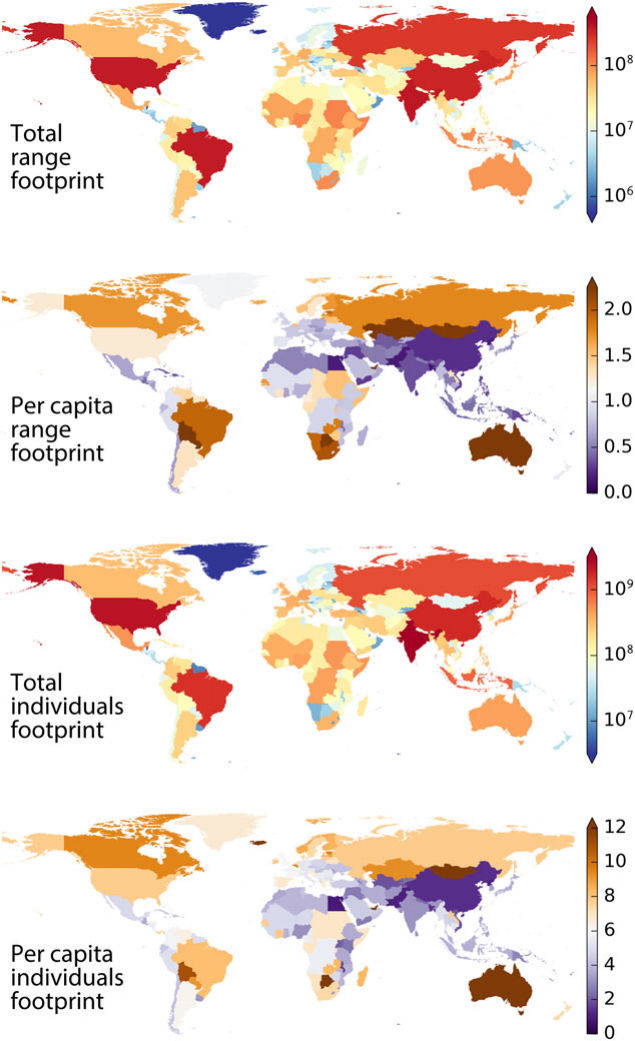
Regional wildlife footprint and per capita regional wildlife footprint, in units of km^2^ of occupied bird ranges and numbers of missing individual birds, medium estimates (see also, Table B1).

The wildlife footprint of a region represents the wildlife loss that occurs anywhere in the world as a result of the purchasing activities of residents of that region (Figure 1, Table B1). Regional wildlife footprints vary greatly by geographic region and are largest in regions with large human populations and large economies, such as the United States, India, China, Brazil, and Russia. Per capita wildlife footprints, representing global wildlife losses due to the average purchasing behavior of an individual resident of a region, range from 0.1 km^2^ (Bangladesh) to 5.1 km^2^ (Mongolia) of occupied ranges and from 0.8 ± 0.5 (Egypt) to 21.1 ± 13.5 (Mongolia) individual birds.

The economic sectors with the largest wildlife footprints are those related to food production and consumption, a pattern that reflects the land use basis of the wildlife footprint calculations (Tables 1 and B2). A global average consumer purchase of US$100 of processed rice drives the downstream occupation of 0.14 km^2^ of bird ranges and the displacement of 1.14 ± 0.50 individual birds for 1 year. Sectors without significant direct land uses, such as insurance or electronics, are responsible for wildlife footprints through their indirect effects on increasing the activity of land intensive sectors from which they purchase goods and services. Per dollar wildlife footprints also vary greatly by region, reflecting variation in production efficiency, baseline wildlife levels in that region, and product prices (Table B3).

**Table 1:** Global average estimates of bird ranges occupied and bird individuals displaced for 1 year due to US $100 of consumer purchases (see also, Tables B2 and B3)

Purchase (US $100)	Occupied range (km^2^)	Missing individuals
Bovine meat products	0.18	0.94 (0.47–1.42)
Dairy products	0.09	0.51 (0.24–0.77)
Processed rice	0.14	1.14 (0.64–1.64)
Sugar	0.04	0.29 (0.15–0.43)
Paper products	0.01	0.03 (0.02–0.05)
Metal products	0.00	0.01 (0.01–0.02)
Electronic equipment	0.00	0.01 (0.01–0.02)
Electricity	0.00	0.01 (0.00–0.01)
Air transport	0.00	0.01 (0.00–0.01)

Footprints are land use based and do not include losses due to climate change or other human impacts.

**Figure 2:**
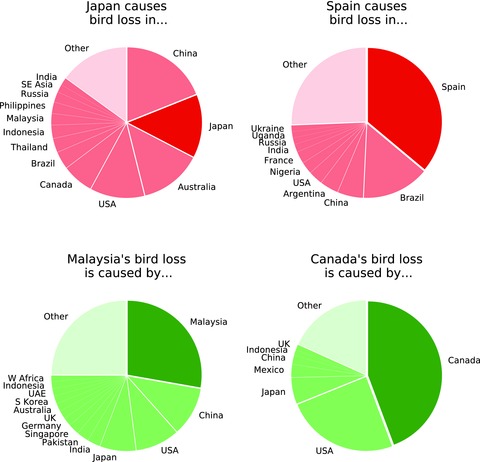
Locations of bird losses, measured in km^2^ of occupied bird ranges, driven by economic consumption in Japan and Spain (globally significant importers), and locations of upstream drivers of local bird losses in Malaysia and Canada (globally significant exporters) (see also, Tables B4–B7).

Approximately 23% of the global wildlife footprint is traded internationally. Three highly integrated regions, Germany, Spain, and France, appear as both significant importers and exporters of embodied wildlife losses (Table 2). Four high‐income regions, Japan, the United Kingdom, Italy, and South Korea, are identified as significant global importers. Australia, Canada, Argentina, Thailand, Viet Nam, Cote d'Ivoire, Malaysia, and Ghana are identified as significant global exporters. Figure 2 shows the locations of bird range occupation driven by two significant importing regions and the drivers of bird range occupation in two significant exporting regions. Additional data on bilateral wildlife footprint trade can be used to identify globally important flows of embodied wildlife losses between individual regions (Tables B4–B7).

**Table 2:** Wildlife footprint, proportion of footprint imported, local losses, and proportion of local losses exported for 15 globally significant importing regions and exporting regions (see also, Tables B4–B7)

	Ranges (million km^2^)				Ranges (million individual birds)						
	Footprint	Import prop.	Local loss	Export prop.	Footprint		Import prop.		Local loss		Export prop.
Japan	77	0.86	–	–	477	(228–726)	0.83	(0.82–0.86)	–	–	–
UK	53	0.81	–	–	362	(160–564)	0.71	(0.69–0.80)	–	–	–
Italy	47	0.77	–	–	320	(147–494)	0.69	(0.68–0.71)	–	–	–
S Korea	28	0.91	–	–	165	(80–251)	0.86	(0.86–0.89)	–	–	–
Germany	68	0.78	25	0.39	442	(191–693)	0.71	(0.69–0.79)	209	(66–352)	0.39
France	52	0.61	32	0.36	377	(157–597)	0.51	(0.48–0.61)	290	(96–485)	0.36
Spain	44	0.64	24	0.34	303	(157–448)	0.53	(0.50–0.54)	217	(120–313)	0.34
Australia	–	–	139	0.42	–	–	–	–	680	(393–967)	0.42
Canada	–	–	91	0.56	–	–	–	–	462	(182–742)	0.54
Argentina	–	–	90	0.46	–	–	–	–	411	(189–633)	0.45
Thailand	–	–	51	0.48	–	–	–	–	526	(299–752)	0.48
Vietnam	–	–	36	0.43	–	–	–	–	209	(117–301)	0.43
Cote d'Ivoire	–	–	28	0.35	–	–	–	–	178	(102–253)	0.35
Malaysia	–	–	24	0.72	–	–	–	–	182	(103–261)	0.72
Ghana	–	–	23	0.44	–	–	–	–	138	(79–196)	0.45

Export proportion is identical for low, medium, and high estimates of bird individuals.

## Discussion

At the level of regions, these wildlife footprint results are broadly consistent with prior analyses of the relationship between biodiversity loss and economic consumption. In particular, the finding that 23% of the global wildlife footprint is traded internationally is similar to prior estimates that 17% of potential species loss is due to agricultural trade (Chaudhary & Kastner 2016) and that 30% of IUCN Red List species threats are due to international trade (Lenzen *et al*. 2012). The significant role of trade in mediating global biodiversity loss highlights the importance of using international consumption‐based analyses to identify the ultimate geographic drivers of biodiversity impacts.

In contrast to the species loss metrics used by Lenzen *et al*. (2012) and Chaudhary & Kastner (2016), the wildlife footprint uses occupied bird ranges and missing individual birds as measures of impact. These prior studies highlight regions with many endemic species, including Madagascar and Papua New Guinea, as significant global exporters of biodiversity impacts. The wildlife footprint, however, suggests that regions with extensive land use change that affects both rare and common species, such as Australia and Canada, are important global exporters of wildlife impacts. Despite differences in the identification of key exporting regions, the three studies identify similar regions as the largest global importers of biodiversity impacts. All three studies suggest that reducing consumption of ecosystem products in the United States, Japan, Germany, South Korea, and France will be critical to the long‐term protection of global biodiversity.

At the level of sectors, the wildlife footprints per dollar presented here are closely related to biodiversity‐focused life cycle assessment (Souza *et al*. 2015; Curran *et al*. 2016; Teixeira *et al*. 2016), which aims to evaluate the biodiversity impacts associated with individual products. Teixeira *et al*. (2016) report recommendations from a consensus meeting of life cycle assessment experts, and two of these recommendations are implemented in this wildlife footprint analysis: the use of HANPP as a measure of intensity and the expansion of biodiversity outcomes to measures beyond species richness. The contribution of global biodiversity and wildlife footprints to life cycle assessment will continue to grow as higher resolution input‐output tables allow for finer distinctions between the footprint intensities of different product classes.

This wildlife footprint analysis is based on a number of data sets and key assumptions that introduce important uncertainties into the results. In the creation of baseline breeding bird range maps, range polygons do not account for “holes” in ranges that are suitable for breeding but may not be occupied at any given time. As such, the occupied ranges reported here should be understood as reflecting the occupation of potential breeding habitat. In the creation of the baseline bird density maps, present‐day measurements of breeding bird abundance in intact land cover classes are presumed to represent the abundance that would occur throughout that land cover class in the absence of human land use and harvest.

For both measures of baseline wildlife levels, this analysis uses maps of HANPP to estimate the fractional loss of wildlife within a grid cell. When combined with the baseline bird range map, fractional HANPP can be interpreted as a dimensionless intensity factor that represents the proportion of a grid cell that is occupied for human uses. When combined with the baseline individual birds map, fractional HANPP has a more direct biological interpretation as the proportion of potentially present birds that are missing, or lost, due to human land uses and harvest (see *Appendix A*).

The multiregional input‐output tables extracted from the GTAP database identify only 57 economic sectors within each geographic region. Although other global input‐output databases are available (Tukker *et al*. 2009, 2013; Wiedmann *et al*. 2011; Lenzen *et al*. 2013), the harmonized sector codes across regions and fine discrimination of agricultural sectors made the GTAP database particularly appropriate for our land use‐based analysis. A comparative analysis of several major input‐output databases, however, has shown that the choice of input‐output tables affects regional carbon footprint estimates for major economies by less than 10% (Moran & Wood 2014). In footprint applications, the accuracy of multiregional input‐output databases is limited by both sector and regional resolution, which leads to problems of homogeneity, as well as by disparities in the collection and standardization of data across regions.

Beyond these limitations, this wildlife footprint analysis is also restricted in scope. First, the analysis accounts only for human impacts due to land use change and harvest. A climate‐based analysis, for example, would find the highest wildlife footprints to be associated with energy‐intensive sectors that lead to the largest emissions of greenhouse gases. An integrated model of land use, climate, and other human impacts could provide a more inclusive measure of impacts while accounting for interactions between these factors and avoiding potential double counting. Second, this analysis examined only wildlife footprints for birds. An occupied range footprint could be readily calculated for other taxa, such as mammals, reptiles, and amphibians, for which global range maps are available.

The results of this wildlife footprint analysis have several implications for the targeting of global conservation investment and intervention. First, the analysis identifies and quantifies the potential leverage associated with small changes in consumption in regions and sectors with the largest wildlife footprints. For example, reducing the wildlife footprint of the United States by 3% would lead to downstream wildlife gains equivalent to restoring all land in Ecuador to a natural state. Convincing a resident of Australia to reduce his or her own footprint by 10% would create four times the global wildlife savings as convincing a resident of Switzerland to do the same. If every resident of the European Union were to reduce spending on bovine meat products by US$1 per year, the displacement of nearly 5 million individual birds, the number found in about 8,000 km^2^ of boreal forest, could be avoided.

Second, global patterns in wildlife footprint trade highlight regions that may be traditionally over‐ or undervalued as targets of global conservation attention. Regions that have large footprints and that import a relatively high proportion of their footprints, for example, are important targets for consumption‐based interventions, as these regions drive large global wildlife losses that are not apparent from the relatively smaller losses occurring within their own borders. Conversely, regions with large local wildlife losses that export a high proportion of these losses may be less fruitful targets for intervention than they initially appear, as wildlife losses in these exporting regions are largely driven by demands elsewhere in the world. If demand in importing nations is not reduced, local reductions in wildlife losses in exporting regions may not lead to global reductions in wildlife losses but instead simply shift wildlife threats to other regions with lower conservation pressure.

While specific conservation efforts have achieved great successes in many locations, efforts of the global public and conservation organizations have not yet been able to halt or reverse widespread declines in global biodiversity. We suggest that a historical focus on intervening at the level of the immediate, proximate causes of biodiversity loss may be an important reason for this lack of success. The wildlife footprint framework presented here provides a concrete means of identifying the ultimate upstream drivers of biodiversity loss, targeting interventions at the level of consumption rather than downstream impact, and more broadly integrating economic considerations into the practice of conservation biology.

## Supporting information


**Figure A1**: Global estimates of (a) baseline bird ranges, in numbers of overlapping ranges, and (b) baseline individual birds, based on medium‐density estimates from Gaston *et al*. 2003, in individual birds/km^2^.
**Table A1**: Estimated breeding bird densities (individuals/km^2^) for each potential vegetation class in Ramankutty *et al*. 1999, drawn from associated land cover classes in Gaston *et al*. 2003.
**Figure A2**: Global estimates of HANPP, as a percentage of NPP_0_, due to (a) all land uses, (b) cropland, (c) pasture and grazing, and (d) forestry.
**Table A2**: GTAP v8 agricultural sectors and associated crop maps from Monfreda *et al*. 2008.
**Table A3**: Comparison of breeding bird density estimates (birds/km^2^) on cropland and pasture as given by Gaston *et al*. 2003 and as calculated using the HANPP‐based approach used in this analysis.

**Table B1**: Local losses and wildlife footprints in units of occupied bird ranges (km^2^) and missing individual birds.
**Table B2**: Total and per dollar wildlife footprint for 57 global economic sectors.
**Table B3**: Total and per dollar wildlife footprint for 57 economic sectors within each of 129 regions.
**Table B4**: Global imports and exports of embodied occupied bird range footprint between 129 regions.
**Table B5**: Similar to Table B4, but for missing individual bird footprints, low estimate.
**Table B6**: Similar to Table B4, but for missing individual bird footprints, medium estimate.
**Table B7**: Similar to Table B4, but for missing individual bird footprints, high estimate.
