# Supplementary material for: Consumption‐Based Conservation Targeting: Linking Biodiversity Loss to Upstream Demand through a Global Wildlife Footprint
Source: Conserv Lett. 2016 Nov 9;10(5):531–8. doi: 10.1111/con4.12321 (PMC5655738; doi:10.1111/con4.12321)
Supplement: Supplementary file 1 — Figure A1: Global estimates of (a) baseline bird ranges, in numbers of overlapping ranges, and (b) baseline individual birds, based on medium‐density estimates from Gaston et al. 2003, in individual birds/km2. Table A1: Estimated breeding bird densities (individuals/km2) for each potential vegetation class in Ramankutty et al. 1999, drawn from associated land cover classes in Gaston et al. 2003. Figure A2: Global estimates of HANPP, as a percentage of NPP0, due to (a) all land uses, (b) cropland, (c) pasture and grazing, and (d) forestry. Table A2: GTAP v8 agricultural sectors and associated crop maps from Monfreda et al. 2008. Table A3: Comparison of breeding bird density estimates (birds/km2) on cropland and pasture as given by Gaston et al. 2003 and as calculated using the HANPP‐based approach used in this analysis. [file CONL-10-531-s001.pdf]

Consumption-based conservation targeting: Linking biodiversity loss to upstream demand through a global wildlife footprint

Appendix A: Supplementary Methods

This document provides additional details on the wildlife footprint calculations described in the main manuscript. All calculations, except where otherwise noted, were performed in Python 3.4.5 using standard scientific packages provided by the Continuum Anaconda distribution.

A1 Temporal considerations in analyzing wildlife losses

Two broad approaches can be used to account for the temporal dimension of wildlife losses due to land use change. A static or stock-based approach defines wildlife loss as occurring only once, at the instant at which a natural land cover is converted to a human-dominated state. This approach implicitly assumes that continuing occupation of transformed land involves no associated continuing wildlife losses, thus ignoring the potential for the recovery and restoration of existing human-used lands. Conversely, an ongoing or flow-based approach defines loss as recurring continuously in time, such that habitat occupation in any time period, regardless of the date of the original conversion of habitat, continues to represent a loss of wildlife. This approach implicitly assumes unlimited potential for recovery and restoration of existing human used lands.

Consider, for example, a plot of forest containing habitat for 100 birds that is cleared for cropland. The static approach allocates a footprint of 100 birds to the initial actor who clears the plot, while any future actor who continues to harvest crops from the plot has a footprint of zero, even though this harvest likely prevents additional birds from recruiting to the plot. Conversely, the ongoing approach allocates a wildlife footprint of 100 lost birds to the actor who harvests the plot at any time following clearing, regardless of when the original plot was harvested.

Both approaches are valid, as would be a hybrid approach mixing the two in a manner akin to depreciation of a capital investment in finance. This analysis uses the ongoing approach, for which losses in any given year can be estimated from maps of global baseline wildlife levels and existing land uses. The static approach would instead require maps of global baseline wildlife levels and land clearing activities.

While conceptual and not physical in nature, the differences between the static and ongoing calculations of wildlife loss are substantial, and comparison of the two approaches will be an important area for future investigation. In general, the ongoing approach will find the largest wildlife losses to be occurring in regions with the largest baseline wildlife levels and the highest intensity of human land use, while the static approach will find the largest wildlife losses to be occurring in regions with the largest baseline wildlife levels and the largest current land conversion activity.

A2 Wildlife footprint metrics

This analysis uses two different metrics to calculate the global wildlife footprint: occupied bird ranges and missing individual birds.

The occupied bird ranges metric is based on the number of present-day breeding bird ranges that overlap a given geographic area. For this estimate, global maps of wild bird ranges in Geodatabase format were obtained from BirdLife International and NatureServe (Bird Species Distribution Maps of the World 2015, v5.0, http://www.birdlife.org/datazone/home). The 17,759 polygon features in this data set were filtered to the 13,235 features with Presence codes of 1 or 2 (Extant or Probably Extant), Origin codes of 1, 2, or 3 (Native, Reintroduced, or Introduced), and Seasonality codes of 1 or 2 (Resident or Breading Season). Each of these features was then converted to a binary raster on a 5 arc minute grid using the GDAL and OGR bindings available for Python 3. The sum of these binary rasters gives a global map of the total number of present-day breeding bird ranges that overlap each grid cell (Figure A1a).

The missing individual birds metric is based on the number of wild breeding birds of any species that would be present in intact climatically driven, potential vegetation cover in a given geographic area. For this estimate, a global 5 arc minute map of potential climatically driven vegetation classes was obtained from Ramankutty & Foley (1999). Each of the 15 vegetation classes was assigned a baseline bird density, in individuals per km^2, drawn from Gaston et al. (2003) and based on published studies of breeding bird density in different land cover classes (Table A1, Figure A1b). Low, Medium, and High estimates from Gaston et al. (2003) were used to generate a range of estimated baseline bird densities.

A3 Wildlife loss factors

The baseline wildlife maps described above were then combined with a map estimating the fractional loss of wildlife occurring in each grid cell. These fractional losses were calculated according to the human appropriation of net primary productivity (HANPP) in each cell (Vitousek et al. 1986; Rojstaczer et al. 2001; Imhoff et al. 2004; Haberl et al. 2007). Following Haberl et al. (2007), HANPP was defined as the ratio of the net primary produc-

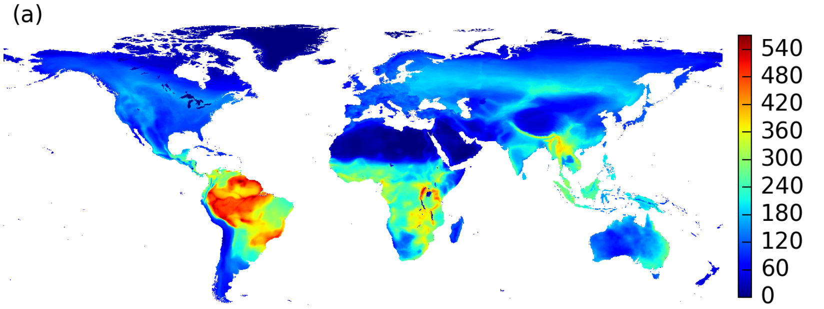

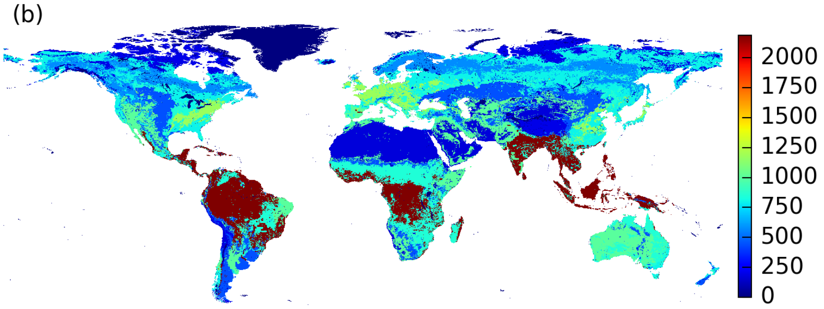

Figure A1: Global estimates of (a) baseline bird ranges, in numbers of overlapping ranges, and (b) baseline individual birds, based on Medium density estimates from Gaston et al. 2003, in individual birds / km^2.

Ramankutty et al. 1999	Gaston et al. 2003	Low	Med	High
Tropical Evergreen Forest/Woodland	Tropical Woodland/Forest	1250	2188	3125
Tropical Deciduous Forest/Woodland	Tropical Woodland/Forest	1250	2188	3125
Temperate Broadleaf Evergreen Forest/Woodland	Temperate Mixed Forest	350	800	1250
Temperate Needleleaf Evergreen Forest/Woodland	Temperate Mixed Forest	350	800	1250
Temperate Deciduous Forest/Woodland	Temperate Deciduous Forest	350	1175	2000
Boreal Evergreen Forest/Woodland	Boreal Forest	150	575	1000
Boreal Deciduous Forest/Woodland	Boreal Forest	150	575	1000
Evergreen/Deciduous Mixed Forest/Woodland	Temperate Mixed Forest	350	800	1250
Savanna	Savanna	500	850	1200
Grassland/Steppe	Grassland/Steppe	100	450	800
Dense Shrubland	Scrubland	600	1000	1400
Open Shrubland	Scrubland	600	1000	1400
Tundra	Tundra	50	200	350
Desert	Hot desert	50	175	300
Polar Desert/Rock/Ice	Ice	0	0	0

Table A1: Estimated breeding bird densities (individuals / km^2) for each potential vegetation class in Ramankutty et al. 1999, drawn from associated land cover classes in Gaston et al. 2003. Tropical Woodland/Forest is the average of the Tropical Forest and Tropical Woodland classes of Gaston.

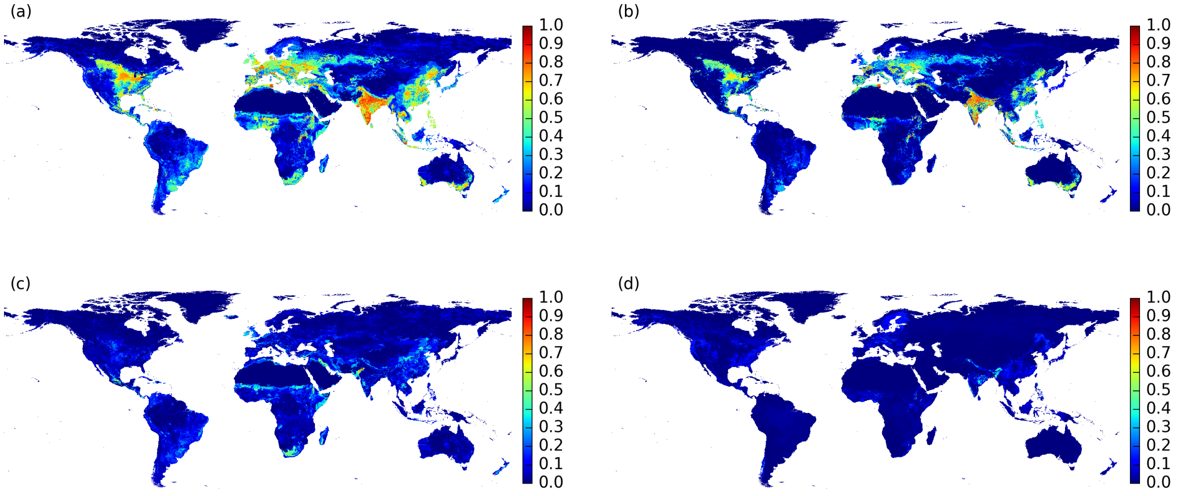

Figure A2: Global estimates of HANPP, as a percentage of NPP_0, due to (a) all land uses, (b) cropland, (c) pasture and grazing, and (d) forestry. Losses due to built up land are locally important but generally not visible on a map at this scale.

tivity that is currently found in a grid cell and the net primary productivity that would be available in a grid cell in the absence of human land use or harvest activities.

The global 5 arc minute HANPP map of Haberl et al. (2007) was disaggregated into four major human land uses: cropland, pasture and grazing, forestry, and built up land (Figure A2). Negative values of HANPP for any land use type and grid cell, which occur when a land use increases the NPP available in a cell, were set to zero, corresponding to zero estimated wildlife loss. Total HANPP was calculated as the sum of the products of each of the four HANPP land use maps and their respective fractional areas in each grid cell (Erb et al. 2007).

When combined with the baseline bird range map, fractional HANPP can be interpreted as a dimensionless intensity factor that represents the proportion of a grid cell that is occupied for human uses. When combined with the baseline individual birds map, fractional HANPP has a more direct biological interpretation as the proportion of potentially present birds that are missing, or lost, due to human land uses and harvest.

This use of HANPP as a measure of proportional loss of bird individuals presumes that total abundance scales linearly with net primary productivity. The empirical shape of this individuals-energy relationship has unfortunately received little attention relative to the better known species-energy relationship (Gaston & Blackburn 2000; Evans et al. 2005; Hurlbert & Jetz 2010), which measures the change in species richness associated with a change in available energy. In the specific case in which a change in available energy is due entirely to change in habitat extent, the individuals-energy relationship is equivalent to a community-level individuals-area relationship. The individuals-area relationship will

be linear when species densities are constant across a grid cell, when species densities are randomly variable within a grid cell, or when habitat loss is randomly placed. While HANPP is due to both habitat loss and habitat degradation, the individuals-energy relationship could thus be expected to be approximately linear in cases where HANPP is driven strongly by habitat loss.

We are not aware of any prior studies of the individuals-energy relationship that examine avian communities, measure abundance at approximately 50–100 km^2 scales, use net primary productivity as a measure of energy, and generalize to a global scale. The closest study to meeting these criteria, by Hurlbert (2004), examined data from the North American Breeding Bird Survey and found a positive relationship between total bird abundance and NDVI in grassland habitats and a negative relationship in deciduous forest. Given the lack of clear empirical evidence, the assumption of a linear relationship between total bird abundance should be taken as tentative and largely based on an appeal to simplicity, represented formally by information theoretic considerations (Jaynes 2003).

A4 Wildlife loss by region and land use

To calculate the bird ranges that are occupied by human activities, the summed range map was multiplied by each of the four land use-specific HANPP maps and the fractional area of each land use per grid cell. These maps was multiplied by a raster giving the area of each 5 arc minute grid cell, created in R 3.3.0 using the *raster* package, producing maps of km^2 of bird ranges lost per grid cell due to each of the four land uses.

To better resolve wildlife losses due to cropland, the map of cropland-driven range losses due was further divided into more finely resolved crop categories. Global 5 arc minute maps of the proportional cell area planted to each of 11 different aggregated crop groups, plus separate maps for rice and wheat, were obtained from Monfreda et al. (2008) (http://www.geog.mcgill.ca/~nramankutty/Datasets/Datasets.html, see also http://www.earthstat.org/data-download/) (Table A2). The total planted area in each grid cell was calculated by summing the planted areas of each of the eleven aggregated crop groups. The eleven crop group maps plus the rice and wheat maps were then used to calculate the planted area for each of nine GTAP v8 agricultural sectors (Table A2, Appendix B).

The planted area map for each sector was then divided by the total planted area map and multiplied by the map of wildlife loss due to cropland. The resulting nine maps, each corresponding to a GTAP v8 agricultural sector, gave the the wildlife loss per grid cell associated with the planting and harvest of each crop sector. Wildlife losses occurring in grid cells without crop group data in Monfreda et al. (2008) were assigned to the nine sectors according to the proportion of total wildlife losses in that GTAP region caused by that crop sector, using loss estimates from grid cells with associated crop group data.

A shapefile of national boundaries from Thematic Mapper (http://thematicmapping

GTAP Sector	Monfreda et al. 2008 crop groups
1 - Paddy rice	Rice
2 - Wheat	Wheat
3 - Cereal grains nec	Cereals - Rice - Wheat
4 - Vegetables fruit nuts	Vegetables&Melons + Fruit + Treenuts
5 - Oil seeds	Oilcrops
6 - Sugar cane sugar beet	SugarCrops
7 - Plant-based fibers	Fiber
8 - Crops nec	Pulses + Roots&Tubers + OtherCrops
9 - Bovine cattle sheep and goats horses	Forage

Table A2: GTAP v8 agricultural sectors and associated crop maps from Monfreda et al. 2008. The wildlife losses due to forage here assigned here to Sector 9 were later divided between Sector 9 and Sector 11 (see text).

.org/downloads/world_borders.php) was downloaded and combined with a crosswalk table (https://www.gtap.agecon.purdue.edu/resources/res_display.asp?RecordID=4328) linking the ISO three letter national alphabetic codes to the 129 GTAP v8 region codes (Appendix B). This map was used to identify and sum the total km^2 of lost ranges occurring in each GTAP region due to each of the nine crop sectors and the three other land use activities (pasture and grazing, forestry, and built up land).

The number of individual birds lost due to human land use activities was calculated in a similar manner to lost bird ranges. Three initial wildlife metric maps, corresponding to the Low, Medium, and High breeding bird density estimates from Gaston et al. (2003), were used to generate a range of bird loss estimates. The final results were in units of bird individuals lost within each GTAP region due to each of the crop groups and land use activities.

A5 Global multi-regional input-output analysis

An environmentally-extended multi-regional input-output analysis (Miller & Blair 2009; Kitzes 2013) was used to link the estimated wildlife losses in each region to the upstream economic activities that drove those losses. A multi-regional input-output table and associated variables, including total output and final demand, were extracted from the Global Trade Analysis Project (GTAP) database v8a (https://www.gtap.agecon.purdue.edu/databases/v8/default.asp) following the procedure described in Peters et al. (2011). The resulting input-output table covers 57 sectors in 129 regions (Appendix B), has a reference year of 2007, and expresses monetary figures in units of 2007 U.S. dollars.

The GTAP database tracks flows between sectors in monetary, not physical terms. Physical input-output tables that instead measure physical resource flows (Hubacek & Giljum

2003; Erb et al. 2009; Haberl et al. 2012) could also be used, but we are not aware of any physical input-output tables with a global scope and complete sectoral coverage. Physical flow accounting is not inherently preferable to monetary accounting. Measurements of physical flows, for example, will not account for the wildlife footprint embodied in international trade in services, such as insurance or financial transactions.

Estimates of wildlife losses due to each of the nine previously identified crop sectors, pasture and grazing, forestry, and built up land within each GTAP region were then allocated to the 57 economics sectors in that region. This initial allocation procedure assigns wildlife losses to the sector within each region that is the proximate cause of those losses. Subsequent calculations using the multi-regional input-output table are used to evaluate the upstream drivers of these downstream, proximate impacts.

Within each region, the the initial allocation was initially completed as follows:

- Cropland: Wildlife losses due to the nine identified crop sectors in Table A2 were assigned to that sector.
- Pasture and grazing: Wildlife losses due to pasture and grazing were allocated to Sector 9, Bovine cattle, sheep and goats, horses.
- Forestry: Wildlife losses due to forestry were allocated to Sector 13, Forestry.
- Built up land: Wildlife losses due to built up land (primarily buildings and roads) were divided amongst all 57 sectors in proportion to the total economic output of that sector.

To complete the initial allocation, the wildlife losses associated with forage crops and grazing, to this point assigned entirely to Sector 9, Bovine cattle, sheep and goats, horses, were divided between Sector 9 and Sector 11, Raw Milk, in proportion to the total economic output of those sectors in that region.

The initial allocations for each of the 57 sectors within each of the 129 regions were combined to create direct requirements vectors, f^d, of length 7353, giving the direct or proximate wildlife loss associated with the activities of each region-sector combination. Four direct input vectors were created, one for the summed range metric and three for the individual birds metrics. The sum of f^d across all 57 sectors within a region gives the total wildlife losses that occur within the borders of that region.

A direct intensity vector, reflecting the wildlife losses directly due to one dollar of output from a given region-sector combination, was then calculated by dividing the direct requirements vector element-wise by a vector of total economic output by region-sector combination, x.

$$F^D = f^d/x \tag{A1}$$

A total intensity vector, F^T, was calculated using Eq. A2.

$$F^T = F^D(\mathbf{I} - \mathbf{A})^{-1} \tag{A2}$$

In Eq. A2, **I** is the identity matrix and **A** is a matrix of input-output coefficients (Miller & Blair 2009). The total intensity vector F^T measures the wildlife footprint-intensity of final consumption in each sector-region combination, equivalent to the total upstream wildlife loss caused by an end consumer spending one dollar to purchase goods or services from that sector within that region.

The total wildlife losses associated with final consumption for a region i was calculated as the element-wise product of F^T and the total final demand by households, government, and industry in that region, c_i.

$$f_i^c = F^T c_i \tag{A3}$$

The final consumption vector f_i^c measures the wildlife loss driven by all sectors in all regions due to the consumption demands of region i. The sum of f_i^c gives the global wildlife loss that occurs due to economic consumption in region i, which is defined as that region's wildlife footprint. The difference between the local wildlife loss in region i, given by the sum of f^d for the 57 sectors of that region, and a region's wildlife footprint is equivalent to net trade in embodied wildlife footprint for that region.

The footprint of a region-sector combination (e.g., United States – Wheat) is calculated as the sum of f_i^c across all regions i for that region-sector combination. A footprint for a sector (e.g., Wheat) is calculated by summing the footprint of the sector-region combination vector across all 129 regions. A footprint intensity for a sector is calculated by dividing a sector's footprint by the summed total output of that sector across all regions.

A6 Validation of individual bird loss estimates

In addition to estimates of breeding bird densities in natural land cover classes, Gaston et al. (2003) also provide estimates of breeding bird density in cropland and pasture. These estimates provide a means of independently checking the method used above to calculate total losses of bird individuals on cropland and pasture.

To perform this check, maps of Low, Medium, and High breeding bird densities in potential vegetation classes were created as described above. For each of cropland and pasture, the *remaining* density of breeding birds in each grid cell on that land use was calculated as the product of this initial density and one minus the HANPP associated with that land use. The total number of global birds on that land use was then calculated as the sum across all grid cells of the product of this remaining density, the fraction of each cell associated with the land use, and the area of that cell. The total area of that land use globally was calculated as the sum across all grid cells of the product of the fraction of each cell associated with

	Crop			Pasture		
	Low	Med	High	Low	Med	High
Gaston et al. 2003	100	300	500	150	375	600
This study	96	189	282	358	699	1041

Table A3: Comparison of breeding bird density estimates (birds / km^2) on cropland and pasture as given by Gaston et al. 2003 and as calculated using the HANPP-based approach used in this analysis. For the method used in this study, Low, Med, and High refer to the three levels of potential vegetation breeding bird densities, drawn from Gaston et al., that were used in concert with the HANPP data to calculate remaining bird densities on cropland and pasture.

the land use and the area of that cell. The average density of birds on that land use was then calculated as the total number of birds globally on that land use divided by the area of that land use.

Table A3 compares the cropland and pasture breeding bird density figures from Gaston et al. (2003) to estimates from the HANPP-based approach used in this analysis. The HANPP-based and Gaston et al. analyses estimate similar breeding bird densities in cropland for the low density potential vegetation map. For the high density potential vegetation map, the HANPP-based analysis estimates approximately 40% fewer birds on world-average cropland, and hence larger bird losses in this land use. For pasture, the HANPP-based analysis estimates approximately twice the breeding bird density given by Gaston et al. For both land uses, the differences between the two approaches are smaller than the differences between the Low and High estimates given for that land use by Gaston et al.

Gaston et al. report a total of 14.66 million km^2 of cropland and 30.98 million km^2 of pasture, while the land use data from Erb et al. (2007) used in the HANPP-based analysis report 15.17 million km^2 of cropland and 46.75 million km^2 of pasture.

As the density estimates in Gaston et al. were based on a semi-qualitative review of published literature, it is difficult to definitively identify the sources of the differences in Table A3. While the HANPP-based analysis is global in scope and proportionally representative of all potential vegetation classes, the data summarized by Gaston et al. are more likely to be biased in scope towards temperate landscapes and other well-studied locations. Conversely, the HANPP-based analysis only accounts for standing NPP in cropland and pasture and does not account for other disturbances in these land uses that might affect total abundance. The HANPP-based analysis also does not directly account for changes in species or community composition in disturbed habitats, factors that are more likely to have been incorporated in the publications underlying the the Gaston et al. estimates.

With regard to the pasture density estimates, much of the discrepancy in estimated bird density may be due to differences in the criteria used to classify land into the “pasture” category. The land use data from Erb et al. (2007) used in the HANPP-based analysis estimate

nearly 50% more global pasture land than Gaston et al., and this additional land is likely to include land of lower intensity use and hence higher remaining breeding bird densities.

A7 Choice of metric for wildlife footprint applications

In applications of the wildlife footprint to policy, management, or communication, units occupied bird ranges or missing individual birds may be used to report wildlife footprints. The allocation of the total global wildlife footprint among regions is very similar under the two metrics (see *Results* and Table B1, Spearman's rank correlation $\rho = 0.97$), reflecting previously observed positive correlations between species diversity and total abundance for birds (Hurlbert 2004). The positive relationship between observed total abundance and species diversity is also the foundation of the "more individuals" or "passive sampling" hypothesis for the origin of the species-area relationship (e.g. Rosenzweig 1995; Gaston 2000; Hurlbert & Jetz 2010).

The choice of metric to use in any particular application will thus be driven by considerations of underlying assumptions and by interpretability. The individual birds metric requires estimates of potentially present birds as well as relatively strong assumptions about the meaning of HANPP-based intensity factors. Conversely, the number of birds present in human-modified habitat is also an quantity that could be measured in the field, while occupied bird ranges does not have an independent means of empirical verification. The unit of missing individual birds is also arguably more intuitively understandable, particularly for communication with the general public.

References

Erb, K.-H., Gaube, V., Krausmann, F., Plutzar, C., Bondeau, A. & Haberl, H. (2007). A comprehensive global 5 min resolution land-use data set for the year 2000 consistent with national census data. *Journal of Land Use Science*, 2, 191–224.

Erb, K.-H., Krausmann, F., Lucht, W. & Haberl, H. (2009). Embodied HANPP: mapping the spatial disconnect between global biomass production and consumption. *Ecological Economics*, 69, 328–334.

Evans, K.L., Warren, P.H. & Gaston, K.J. (2005). Species–energy relationships at the macroecological scale: a review of the mechanisms. *Biological Reviews*, 80, 1–25.

Gaston, K.J. (2000). Global patterns in biodiversity. *Nature*, 405, 220–227.

Gaston, K.J. & Blackburn, T.M. (2000). *Pattern and process in macroecology*. Blackwell Science, Ltd., Malden, MA.

Gaston, K.J., Blackburn, T.M. & Klein Goldewijk, K. (2003). Habitat conversion and global avian biodiversity loss. *Proceedings of the Royal Society B*, 270, 1293–1300.

Haberl, H., Erb, K.H., Krausmann, F., Gaube, V., Bondeau, A., Plutzar, C., Gingrich, S., Lucht, W. & Fischer-Kowalski, M. (2007). Quantifying and mapping the human appropriation of net primary production in earth's terrestrial ecosystems. *Proceedings of the National Academy of Sciences*, 104, 12942–12947.

Haberl, H., Steinberger, J.K., Plutzar, C., Erb, K.-H., Gaube, V., Gingrich, S. & Krausmann, F. (2012). Natural and socioeconomic determinants of the embodied human appropriation of net primary production and its relation to other resource use indicators. *Ecological Indicators*, 23, 222–231.

Hubacek, K. & Giljum, S. (2003). Applying physical input-output analysis to estimate land appropriation (ecological footprints) of international trade activities. *Ecological Economics*, 44, 137–151.

Hurlbert, A.H. (2004). Species-energy relationships and habitat complexity in bird communities. *Ecology Letters*, 7, 714–720.

Hurlbert, A.H. & Jetz, W. (2010). More than "more individuals": the nonequivalence of

area and energy in the scaling of species richness. *The American Naturalist*, 176, E50–E65.

Imhoff, M.L., Bounoua, L., Ricketts, T., Loucks, C., Harriss, R. & Lawrence, W.T. (2004). Global patterns in human consumption of net primary production. *Nature*, 429, 870–873.

Jaynes, E.T. (2003). *Probability Theory: The Logic of Science*. Cambridge University Press, Cambridge, United Kingdom.

Kitzes, J. (2013). An introduction to environmentally-extended input-output analysis. *Resources*, 2, 489–503.

Miller, R. & Blair, P. (2009). *Input-output Analysis: Foundations and Extensions*. Second. Cambridge University Press, Cambridge, United Kingdom.

Monfreda, C., Ramankutty, N. & Foley, J.A. (2008). Farming the planet: 2. Geographic distribution of crop areas, yields, physiological types, and net primary production in the year 2000. *Global Biogeochemical Cycles*, 22, 1–19.

Peters, G.P., Andrew, R. & Lennox, J. (2011). Constructing an environmentally-extended multi-regional input–output table using the GTAP database. *Economic Systems Research*, 23, 131–152.

Ramankutty, N. & Foley, J. (1999). Estimating historical changes in global land cover: croplands from 1700 to 1992. *Global Biogeochemical Cycles*, 13, 997–1027.

Rojstaczer, S., Sterling, S.M. & Moore, N.J. (2001). Human appropriation of photosynthesis products. *Science*, 294, 2549–2552.

Rosenzweig, M.L. (1995). *Species Diversity in Space and Time*. Cambridge University Press, Cambridge, United Kingdom.

Vitousek, P.M., Ehrlich, P.R., Ehrlich, A.H. & Matson, P.A. (1986). Human appropriation of the products of photosynthesis. *BioScience*, 36, 368–373.
